# Long non-coding RNA SNHG1 indicates poor prognosis and facilitates disease progression in acute myeloid leukemia

**DOI:** 10.1242/bio.046417

**Published:** 2019-10-15

**Authors:** Ming Tian, Wanjun Gong, Jingming Guo

**Affiliations:** 1 Department of Hematology, The First College of Clinical Medical Science, China Three Gorges University, Yichang Central People's Hospital, Yichang, Hubei 443000, China; 2 Department of Gastrointestinal Surgery, The First College of Clinical Medical Science, China Three Gorges University, Yichang Central People's Hospital, Yichang, Hubei 443000, China

**Keywords:** lncRNA, SNHG1, Acute myeloid leukemia, Apoptosis, miR-101

## Abstract

The role of long non-coding RNAs (lncRNAs) in acute myeloid leukemia (AML) is becoming increasingly questioned. Previous studies have reported that the lncRNA small nucleolar RNA host gene 1 (SNHG1) is involved in multiple human malignant tumors, while its expression and role in AML is still unexplored. Here, we show that SNHG1 is highly expressed in AML specimens from non-M3 patients, as well as AML cell lines. Meanwhile, upregulation of SNHG1 is correlated with poor prognosis. Notably, SNHG1 facilitates the proliferation and inhibits the apoptosis of AML cells *in vitro*. Consistent with these findings, knockdown of SNHG1 significantly inhibits AML progression in an immunodeficient mouse model. Mechanistically, we found that an anti-tumor microRNA-101 (miR-101) is upregulated and its target genes are downregulated in AML cells after SNHG1 knockdown. Further investigations display that SNHG1 can serve as a competing endogenous RNA to inhibit miR-101. In conclusion, our data indicate that SNHG1 plays an important role in facilitating AML progression at least in part by negatively regulating miR-101, and provides a new target for treating AML.

## Abstract (precis)

**Summary:** Long non-coding RNA SNHG1 can facilitate the progression of acute myeloid leukemia by targeting an anti-tumor miR-101, and the upregulation of SNHG1 can predict poor prognosis in acute myeloid leukemia.

## INTRODUCTION

Acute myeloid leukemia (AML) is a type of hematologic malignant disease characterized by abnormal proliferation of primitive and juvenile myeloid cells in bone marrow (BM) and peripheral blood (PB), and significant suppression of normal hematopoiesis (Burnett and Venditti, 2006; Marcucci et al., 2011; Thomas and Majeti, 2017). AML is well known for its high incidence, recurrence and death rates (Kadia et al., 2016). Although many strategies, such as chemotherapy, supportive therapy and hematopoietic stem cell (HSC) transplantation, have been applied to treat AML, the prognosis of this disease is still poor (Burnett et al., 2011; Marcucci et al., 2011). Therefore, there is an urgent need to understand its intrinsic molecular mechanisms and then develop effective measures for diagnoses and treatment of AML.

Previous studies have confirmed that gene abnormalities contribute to the development and progression of AML (Bullinger et al., 2017). At present, growing evidence indicates that epigenetic dysregulations including acetylation, DNA methylation and non-coding RNAs (ncRNAs) are responsible for this disease (Zhang and Tao, 2019). Long non-coding RNAs (lncRNAs) are a kind of ncRNA with a length over 200 nucleotides that participate in various physiological and pathological processes by transcriptional and post-transcriptional regulation of gene expression (Beermann et al., 2016; Quinn and Chang, 2016). Recently, lncRNAs have been getting more and more attention due to their role in promoting the development and progression of many kinds of malignant tumors, including leukemia (Ma et al., 2018; Quinn and Chang, 2016). So far, several lncRNAs, such as LINC00265, LINC00152, ANRIL, H19 and NEAT1, have been explored in AML (Ma et al., 2018; Sun et al., 2018; Zhang et al., 2018; Zhang and Tao, 2019; Zhao et al., 2019). However, the underlying function, molecular mechanism and clinical significance of lncRNAs in AML have not been fully uncovered.

Small nucleolar RNA host gene 1 (SNHG1), located on chromosome 11, is a novel lncRNA transcribed from U22 host gene (*UHG*) (Thin et al., 2019). Studies have reported that SNHG1 can promote cell proliferation, invasion and metastasis, and inhibit cell apoptosis in multiple human cancers such as esophageal, liver, gastric, lung, colorectal and prostate cancer (Li et al., 2018; Thin et al., 2019; Xu et al., 2018). Some researchers even recommend SNHG1 as a prognostic biomarker for tumors (Xiao et al., 2018). Of note, it was reported that SNHG1 is markedly upregulated in runt-related transcription factor 1 (*RUNX1*) mutation-induced AML (Garzon et al., 2014). However, the exact role of SNHG1 in AML remains unknown.

In this study, we first found that SNHG1 is highly expressed in AML cell lines as well as in specimens from non-M3 AML patients. Notably, upregulation of SNHG1 is correlated with poor prognosis and survival rate. Further investigations reveal that SNHG1 contributes to the progression of AML by negative regulation of a tumor suppressor, miR-101. Collectively, our findings demonstrate that SNHG1 is an oncogene and can act as a therapeutic target for AML.

## RESULTS

### SNHG1 is highly expressed in AML specimens and cell lines and upregulation of SNHG1 is correlated with poor prognosis

**Fig. 1.**
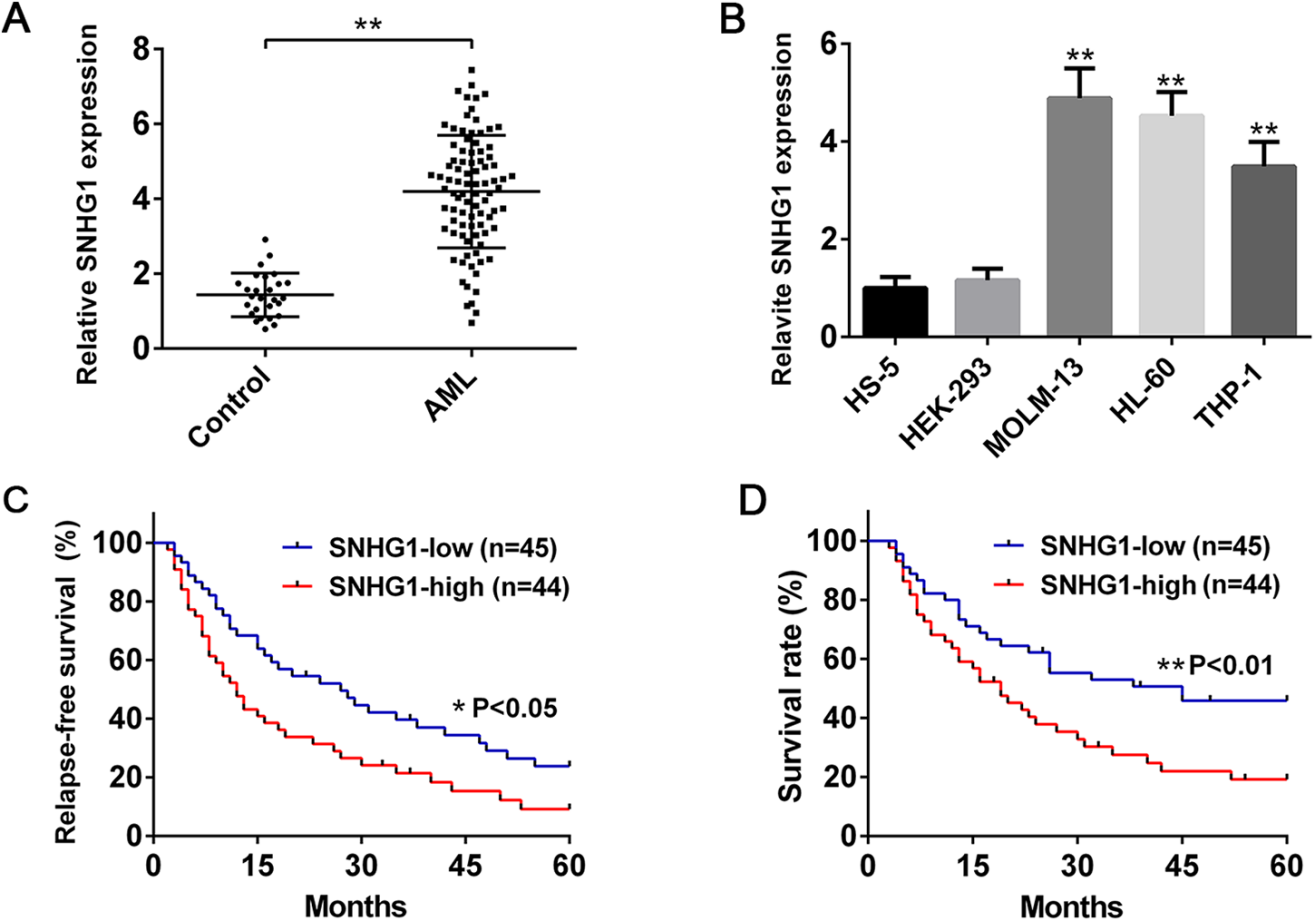
**SNHG1 is highly expressed in AML specimens and cell lines and upregulation of SNHG1 is correlated with poor prognosis.** (A) qPCR analysis of the relative expression of SNHG1 in BM specimens from 89 non-M3 AML patients and 27 healthy controls. (B) qPCR analysis of the relative expression of SNHG1 in HS-5, HEK-293, MOLM-13, HL-60 and THP-1 cells (*n*=6). (C,D) AML patients were divided into two groups (high SNHG1 expression group and low SNHG1 expression group) based on the median expression levels. (C) Relapse-free survival (RSF) time and (D) overall survival rates of AML patients with high or low SNHG1 expression are showed as Kaplan–Meier curves (log-rank test test). *P<0.05, **P<0.01.

To evaluate whether SNHG1 is implicated in AML, we first collected the BM specimens from AML patients and healthy controls. It was found that SNHG1 was more highly expressed in the BM from non-M3 AML patients than healthy control by qPCR analysis (Fig. 1A). In addition, we also observed that the expression of SNHG1 is high in AML cell lines (MOLM-13, HL-60 and THP-1) (Fig. 1B), suggesting that SNHG1 may play a critical role in AML pathogenesis.

We then analyzed the correlation between SNHG1 expression and clinicopathological characteristics in AML patients, and found that high SNHG1 expression was significantly related to higher white blood cell (WBC) count, unfavorable cytogenetics, unfavorable European LeukemiaNet (ELN) risk stratification (Döhner et al., 2017), lower complete remission (CR) rate and higher relapse rate (Table 1 and Table S1). However, there was no significant association of SNHG1 expression with other clinical features including age, gender, BM blasts and of French–American–British (FAB) classification (Table 1). Importantly, AML patients with high expression of SNHG1 had shorter relapse-free survival (RSF) time and lower overall survival rate compared with those with low expression of SNHG1 (Fig. 1C,D). Furthermore, multivariate analysis showed that SNHG1 expression was an independent predictive factor for overall survival in AML patients (Table 2). Thus, SNHG1 may act as a potential prognostic biomarker of AML, with upregulation of SNHG1 indicating an adverse clinical outcome.

**Table 1.**
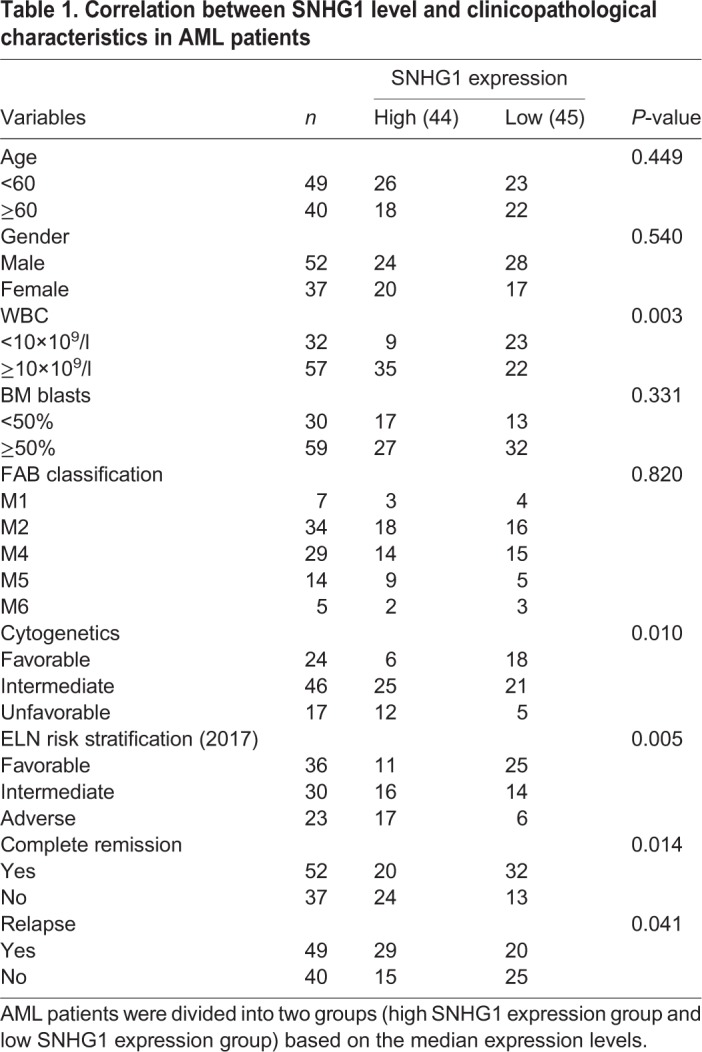
**Correlation between SNHG1 level and clinicopathological characteristics in AML patients**

**Table 2.**
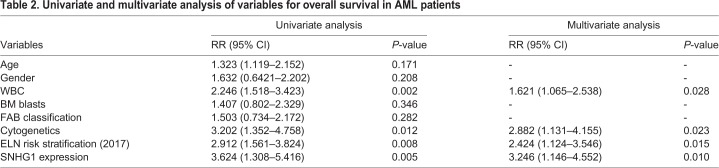
**Univariate and multivariate analysis of variables for overall survival in AML patients**

### SNHG1 facilitates proliferation and cell cycle progression, and inhibits apoptosis of AML cells

**Fig. 2.**
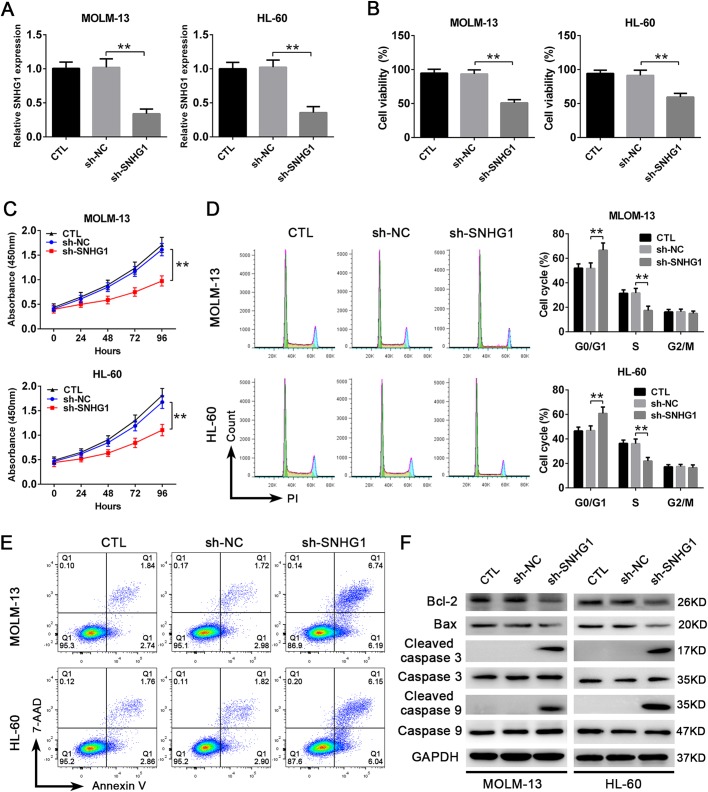
**SNHG1 facilitates the proliferation and cell cycle progression and inhibits the apoptosis of AML cells.** (A) The reduced expression of SNHG1 in MOLM-13 and HL-60 cells after being transduced with lentivirus expressing shRNA against SNHG1 (sh-SNHG1) or scrambled shRNA (sh-NC) (*n*=6). (B) The viability of MOLM-13 and HL-60 cells after knockdown of SNHG1**,** determined by Trypan Blue exclusion (*n*=6). (C) The proliferation of MOLM-13 and HL-60 cells after knockdown of SNHG1, detected by CCK-8 assay (*n*=6). (D) Cell cycle analysis of HL-60 and MOLM-13 cells after knockdown of SNHG1, measured by flow cytometry (*n*=6). (E) The apoptosis of MOLM-13 and HL-60 cells after knockdown of SNHG1, determined by flow cytometry (*n*=6). (F) Western blots detecting the expressions of anti-apoptotic and pro-apoptotic proteins in MOLM-13 and HL-60 cells after knockdown of SNHG1 (*n*=6). **P<0.01.

To further determine the role of SNHG1 in AML, we next used a lentivirus carrying small hairpin RNA (shRNA) to knock down SNHG1 expression in AML cells. As shown in Fig. 2A, the efficiency of knockdown was verified by qPCR. Then we found that, compared with the sh-NC group, transfection of sh-SNHG1 significantly decreased the viability of AML cells (Fig. 2B). Additionally, Cell Counting Kit-8 (CCK-8) assay showed that knockdown of SNHG1 evidently suppressed the proliferation of AML cells (Fig. 2C). Consistent with these findings, cell cycle analysis using flow cytometry showed that knockdown of SNHG1 significantly impeded cell cycle progression in AML cells, with a majority of cells arrested in G0/G1 phases (Fig. 2D). On the other hand, we observed an increase in the apoptosis of AML cells after SNHG1 knockdown (Fig. 2E). Western blotting analysis showed that the expression of anti-apoptotic protein, Bcl-2, was decreased and the expressions of pro-apoptotic proteins (Bax, Cleaved Caspase 3 and Cleaved Caspase 9) were increased (Fig. 2F). Altogether, these results illustrate that SNHG1 is involved in promoting the proliferation and inhibiting the apoptosis of AML cells.

### SNHG1 promotes the progression of AML *in vivo*

**Fig. 3.**
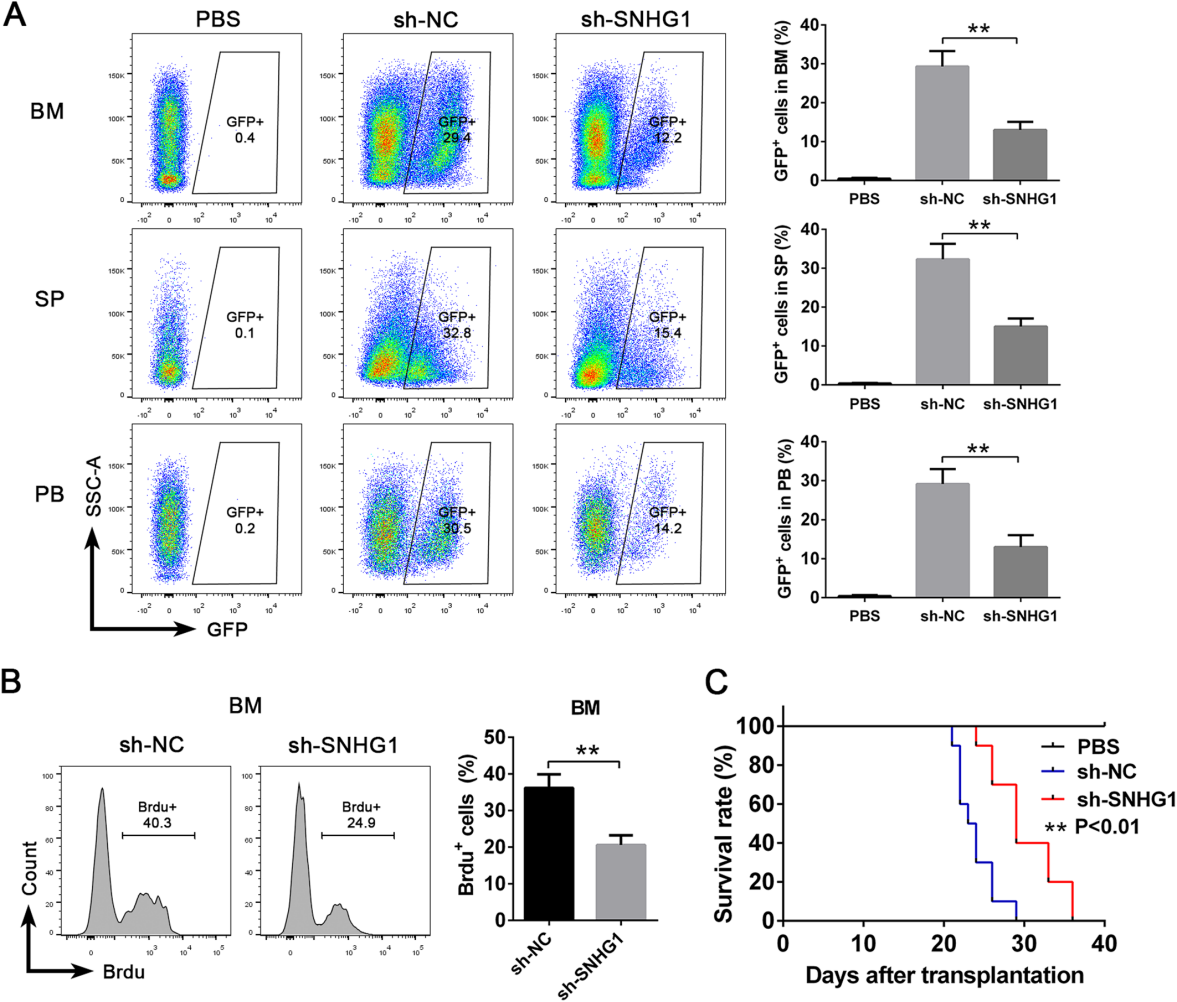
**SNHG1 promotes the progression of AML *in vivo*.** (A,B) Immunodeficient mice (NOD/SCID) were intravenously injected with PBS, or AML cells transduced with sh-NC and sh-SNHG1. At 3 weeks after transplantation, (A) the percentages of GFP^+^ cells in recipients' BM, SP and PB were analyzed by flow cytometry, and (B) the proliferation of GFP^+^ cells in the BM were measured by *in vivo* Brdu incorporation assay (*n*=6 mice per group). (C) The survival rates of mice intravenously injected with PBS, or AML cells transduced with sh-NC and sh-SNHG1 (*n*=10 mice per group). **P<0.01.

To further confirm the function of SNHG1 *in vivo*, we used a xenotransplantation model. AML cells transduced with sh-NC or sh-SNHG1 were transplanted into immunodeficient mice (NOD/SCID mice) by tail vein injection. Three weeks after transplantation, we observed that knockdown of SNHG1 resulted in a significant decrease in the percentage of GFP^+^ cells in the BM, spleen (SP) and peripheral blood (PB) obtained from recipient mice by flow cytometric analysis (Fig. 3A). Meanwhile, an *in vivo* 5-bromodeoxyuridine (Brdu) incorporation assay displayed that the proliferation of GFP^+^ AML cells from the sh-SNHG1 group was more decreased than in the control group after transplantation (Fig. 3B). Furthermore, the mice transplanted with AML cell-transduced sh-SNHG1 survived longer than the control mice (Fig. 3C). These results suggest that SNHG1-knockdown could inhibit the progression of AML *in vivo*.

### SNHG1 promotes the progression of AML by negatively regulating miR-101

**Fig. 4.**
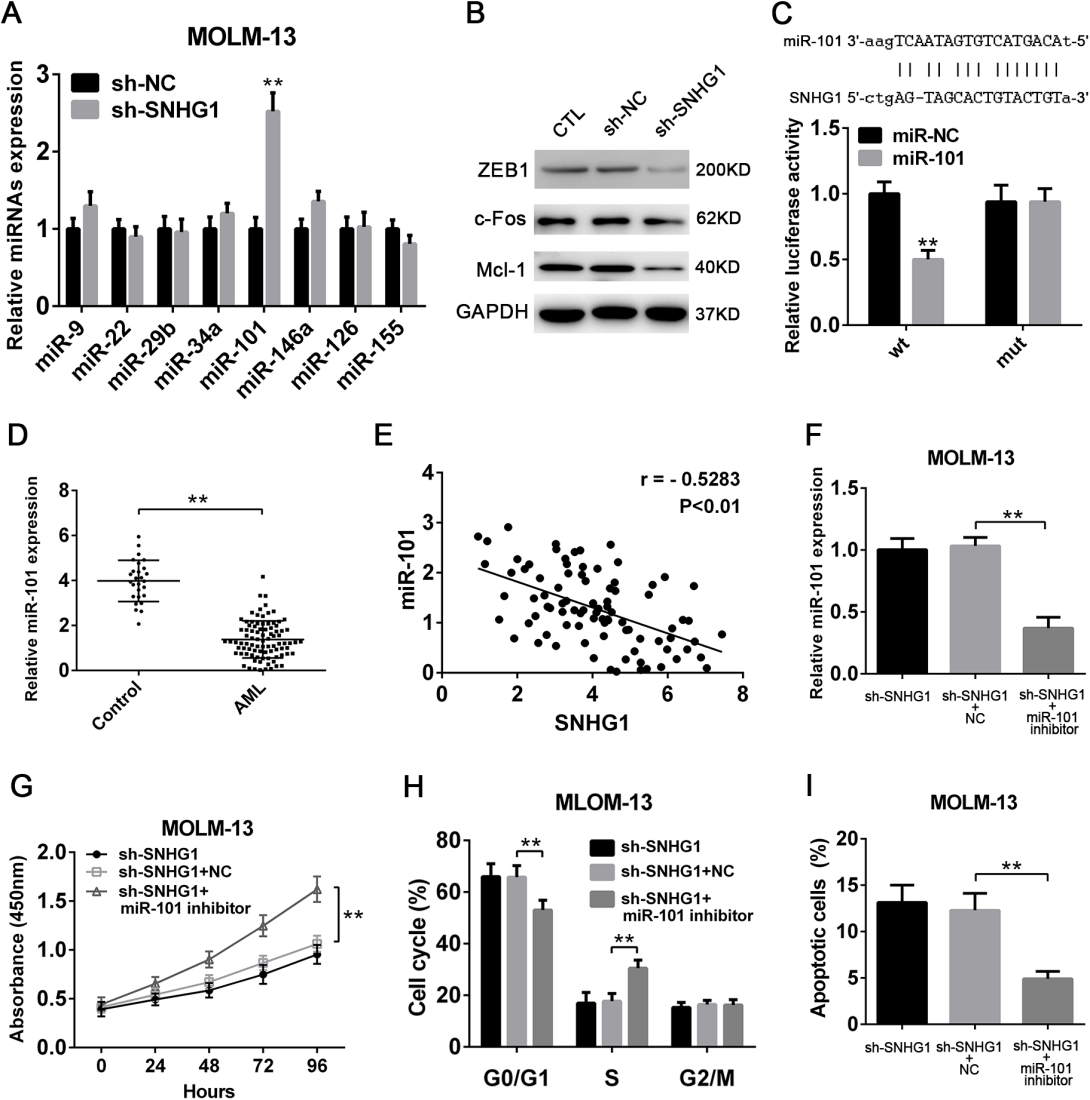
**SNHG1 promotes the progression of AML by negatively regulating miR-101.** (A) The relative expressions of miR-9, miR-22, miR-29b, miR-34a, miR-101, miR-126, miR-146a and miR-155 in MOLM-13 cells after knockdown of SNHG1 (*n*=6). (B) Western blotting detecting the expressions of c-Fos, ZEB1 and Mcl-1 in MOLM-13 cells after knockdown of SNHG1. (C) Relative luciferase activity in HEK-293 cells after co-transfected with wild-type (wt) or mutant (mut) SNHG1 and miR-101 or control miRNA, determined by the luciferase reporter assay (*n*=6). (D) qPCR analysis of the relative expression of miR-101 in BM specimens from 89 non-M3 AML patients and 27 healthy controls. (E) Pearson correlation analysis showing the significantly negative relationship between miR-101 and SNHG1 expression in specimens from 89 non-M3 AML patients (r=−0.4647, *P*<0.01). (F–I) MOLM-13 cells with or without knockdown of SNHG1 were transfected with miR-101 inhibitor or negative control (NC). Then, (F) the expression of miR-101 was analyzed by qPCR. Meanwhile, the (G) proliferation was measured by the CCK-8 assay, and the (H) cell cycle and (I) apoptosis were detected by flow cytometry (*n*=6). **P<0.01.

Next, we set out to explore the underlying molecular mechanism by which SNHG1 regulates the pathogenesis of AML. Given that lncRNAs can function as a competing endogenous RNA to sponging microRNAs (miRNAs), we measured the expressions of several miRNAs that have been reported to be related to leukemia aggressiveness (Chen et al., 2013; Lechman et al., 2016; Mims et al., 2013; Mohr et al., 2017; Pulikkan et al., 2010; Shen et al., 2016) after SNHG1 knockdown. It was found that miR-101, an anti-tumor miRNA, was markedly upregulated in AML cells when SNHG1 was knocked down (Fig. 4A,B; Fig. S1A,B). Meanwhile, the recognized target genes of miR-101, such as *c-Fos*, *ZEB1* and *Mcl-1*, known to promote tumor growth (Gonzales-Aloy et al., 2019; Pan et al., 2015; Stavropoulou et al., 2016), were significantly decreased in AML cells transduced with sh-SNHG1 (Fig. 4B). Further, bioinformatic predictions revealed that SNHG1 can directly bind miR-101, which was confirmed by the luciferase reporter assay (Fig. 4C). Indeed, the expression of miR-101 was distinctly downregulated in BM specimens from AML patients (Fig. 4D). In particular, miR-101 expression was negatively correlated with SNHG1 levels (Fig. 4E). These findings indicate that SNHG1 may negatively regulate miR-101 in AML cells. Finally, to validate whether the decrease of miR-101 is responsible for the effect of SNHG1 in AML, we used a miR-101 inhibitor. Specifically, we found that suppression of miR-101 in AML cells significantly abrogated reduced proliferation, impeded cell cycle progression and increased apoptosis induced by SNHG1 knockdown (Fig. 4F–I; Fig. S1C–F). Collectively, our data demonstrate that SNHG1 is able to promote the progression of AML, at least in part, through negative modulation of anti-tumor miR-101.

## DISCUSSION

AML is a complex malignant disease with high heterogeneity in biology and etiology (Marcucci et al., 2011; Thomas and Majeti, 2017; Xu et al., 2016). Although a lot of excellent research about AML has been done, the pathogenesis is still obscure and the clinical outcome is still not very satisfactory (Khalife et al., 2015; Sun et al., 2018). LncRNAs, previously identified as the ‘noise’ in genomes, have been confirmed to be involved in various biological processes, especially in cancers including leukemia (Garzon et al., 2014; Quinn and Chang, 2016). In the present study, we show for the first time that lncRNA SNHG1 plays an important role in the development and progression of AML.

With the development of sequencing technology and bioinformatics, many lncRNAs have identified associated with the occurrence and development of tumors (Feng et al., 2018). Recently, several lncRNAs, including ANRIL, H19 and NEAT1, have been found to affect disease progression in AML (Sun et al., 2018; Zhang et al., 2018; Zhao et al., 2019). In addition, studies have shown that SNHG1 is frequently overexpressed in multiple solid tumors (Thin et al., 2019), while its expression pattern in AML has not been uncovered. Here, we found that SNHG1 is significantly upregulated in BM from non-M3 AML patients, hinting that SNHG1 may be involved in AML progression. However, unlike SNHG5 (Li and Sun, 2018), we did not find a significant relationship between SNHG1 expression and FAB classification. Further, we observed that AML patients with high expression of SNHG1 had poorer prognosis, which is consistent with our speculation. Knowing that expression of SNHG1 is non-specific in AML, we do not recommend SNHG1 as a diagnostic biomarker. However, our data suggest that SNHG1 can function as a candidate prognostic marker for AML.

Tumor cells were characterized by abnormal proliferation and reduced apoptosis. It has been reported that SNHG1 can affect the growth of many kinds of tumor cells (Thin et al., 2019; Xiao et al., 2018), whereas the precise role of SNHG1 in AML have not been clarified. In the present study, we show that knockdown of SNHG1 inhibits the proliferation and cell cycle progression, and accelerates the apoptosis of AML cells *in vitro*. Importantly, SNHG1 inhibits the apoptosis of AML cells, probably by upregulating Bcl-2 and downregulating Bax and Cleaved Caspase 3 and Cleaved Caspase 9, which is in accordance with a previous study (Zhao et al., 2015). Furthermore, we confirmed that SNHG1 knockdown also progresses AML *in vivo*. Hence, our data demonstrate that SNHG1 may play an oncogenic role in AML.

Previous research has reported that SNHG1 can regulate several molecules or signaling pathways (Thin et al., 2019; Xiao et al., 2018). For example, SNHG1 affects colorectal cancer cell growth via regulation of EZH2 and miR-154-5p (Li et al., 2019). Moreover, SNHG1 suppresses pancreatic cancer cell proliferation, invasion and metastasis through inhibiting the Notch-1 signaling pathway (Cui et al., 2019). Besides, SNHG1 accelerates the deterioration of non-small cell lung carcinoma (NSCLC) by negative modulation of miR-497 (Li et al., 2018). In our work, we found that knockdown of SNHG1 leads to an increase in the expression of miR-101, a tumor suppressor. It is well established that miRNAs play a central role in the regulation of cell behaviors by post-transcriptional regulation of gene expression (Barrera-Ramirez et al., 2017; Havelange et al., 2011; Trino et al., 2018). Then, we observed that miR-101 target genes (*c-Fos, ZEB1* and *Mcl-1*) known to promote cancer progression were significantly downregulated in AML cells with SNHG1 knockdown. Thus, Mcl-1 and c-Fos may be also responsible for SNHG1-mediated inhibition of AML cell apoptosis. Subsequent investigations reveal that SNHG1 can act as a sponge to bind miR-101, eventually resulting in the progression of AML, which is consistent with a previous study (Deng et al., 2018). Therefore, targeting SNHG1 may be an optional strategy for treatment of AML or other types of cancers. However, we cannot rule out the possibility that there are other mechanisms by which SNHG1 regulates the behavior of AML cells. On the other hand, the current view is that AML is a malignant myeloid hematopoietic stem/progenitor cell disease (Burnett and Venditti, 2006; Lechman et al., 2016; Thomas and Majeti, 2017). One of the main reasons behind the poor prognosis and high relapse is the persistence of leukemia stem cells (LSCs) (Lechman et al., 2016; Stavropoulou et al., 2016). Thus, whether SNHG1 regulates LSC function still needs further research.

In conclusion, our study highlights the significance of SNHG1 in AML progression by sponging miR-101, therefore providing a new method for AML treatment and prognosis evaluation. However, more efforts are required to further uncover the role and underlying mechanisms of AML-specific lncRNAs.

## MATERIALS AND METHODS

### Patients and specimens

A total of *de novo* 89 non-M3 AML patients (without any other type of malignancy) and 27 healthy volunteers from Yichang Central People's Hospital were recruited in this study. The AML patients were diagnosed according to the criteria of FAB and the 2016 World Health Organization (WHO) classification (Döhner et al., 2017). All patients included in the study received Standard AML therapy following the protocol provided from the Dutch–Belgian Hematology–Oncology Cooperative Group. BM specimens were obtained from all participants that provided written informed consent. The study was approved by the Ethics Committee of the Yichang Central People's Hospital.

### Cell lines and culture

Human AML (HL-60 and THP-1) and human embryonic kidney cell lines (HEK-293) were obtained from Cell Bank of Chinese Academy of Sciences (Shanghai, China). Human AML line MOLM-13 and human bone marrow stromal cell (HS-5) were purchased from BeNa Culture Collection (Beijing, China). HL-60, THP-1, MOLM-13 and HS-5 cells were grown in RPMI-1640 Medium (HyClone, South Logan, UT, USA) containing 10% fetal bovine serum (FBS; Gibco, Thermo Fisher Scientific, Waltham, MA, USA). HEK-293 cells were cultured in DMEM medium (high glucose; Gibco) supplemented with 10% FBS. All cells were cultured at 37°C in a humidified atmosphere of 5% CO_2_.

### Cell transfection

The lentiviruses carrying shRNA against SNHG1 (sh-SNHG1) or scrambled shRNA (sh-NC) or were purchased from GenePharma Co., Ltd (Shanghai, China). The sequence of sh-SNHG1 was: GGTTTGCTGTGTATCACATTTCTCGAGAAATGTGATACACAACCTTTT (Xu et al., 2018). miR-101 inhibitor and negative control (NC) were obtained from RiboBio Co., Ltd (Guangzhou, China). The lentiviruses were transduced using polybrene (GenePharma Co., Ltd), and the miR-101 mimic, inhibitor and the corresponding NC were transfected using lipofectamine 3000 reagent (Invitrogen, Carlsbad, CA, USA) according to the manufacturer's instructions.

### Cell viability assay

This assay was performed using a Trypan Blue Staining Cell Viability Assay Kit (Beyotome Biotechnology, Beijing, China). 5 min after staining with Trypan Blue, cell viability was determined by directly counting under the microscope (Carl Zeiss, Jena, Germany).

### CCK-8 assay

In brief, cells (3×10^3^/well) were seed into 96-well plates. After culturing for 0, 24, 48, 72 or 96 h, each well had 10 μl of CCK-8 solution (Dojindo, Kumamoto, Japan) added. Then, cells were incubated at 37°C for an additional 2 h, and the absorbance at 450 nm was measured on a microplate reader (Thermo Fisher Scientific).

### Cell cycle and apoptosis assays

For cell cycle assay, cells were fixed by 75% ethanol for 24 h. After washing with phosphate buffer saline (PBS), cells were treated with RNase and stained with Propidium Iodide (PI). Finally, samples were detected on a FACSCanto flow cytometer (BD Biosciences, San Jose, CA, USA). For apoptosis assay, cells were washed twice with cold PBS. Then, cell apoptosis were measured using the Annexin V-FITC/7-AAD Kit (BD Biosciences) according to the manufacturer's instructions. All data were analyzed using FlowJo7.6 software (TreeStar, San Carlos, CA, USA).

### qPCR

Total RNA from BM cells and cultured cells were extracted using TRIzol reagent (Invitrogen, Carlsbad, USA). For detection of SNHG1 (NCBI accession number: NR_152575.1), cDNA was synthesized by a PrimeScript RT reagent kit (Takara, Tokyo, Japan) and qPCR was performed by a SYBR Premix Ex Taq kit (Takara) following the manufacturer's guidelines. Data were normalized to *GAPDH*. Primers used were as follows: *SNHG1* (forward, 5′-ACGTTGGAACCGAAGAGAGC-3′ and reverse, 5′-GCAGCTGAATTCCCCAGGAT-3′), *GAPDH* (forward, 5′-CACCCACTCCTCCACCTTTGA-3′ and reverse, 5′-CCTGTTGCTGTAGCCAAATTCG-3′). For detection of miRNA, cDNA was synthesized by a miRcute Plus miRNA Fist-Strand cDNA Kit (TianGen Biotech, Beijing, China) and qPCR was conducted by a miRcute Plus miRNA qPCR Detection Kit (TianGen Biotech) according to the manufacturer's instructions. Data were normalized to U6 snRNA. The primers used for detecting miRNAs and U6 were purchased from TianGen Biotech. All qPCR assays were carried out on a CFX96™ Real-Time system (BioRad, Hercules, CA, USA).

### Western blotting

Western blotting assay was performed as described previously (Ma et al., 2018). The primary antibodies against human Bcl-2 (#4223S, dilution 1/1000; Cell Signaling Technology), Bax (#5023S, dilution 1/1000; Cell Signaling Technology), Cleaved Caspase 3 (#ab2302, dilution 1/1000; Abcam), Caspase 3 (#ab197202, dilution 1/1000; Abcam), Cleaved Caspase 9 (#ab2324, dilution 1/1000; Abcam), Caspase 9 (#ab219590, dilution 1/1000; Abcam), ZEB1 (#3396S, dilution 1/1000; Cell Signaling Technology), c-Fos (#2250S, dilution 1/1000; Cell Signaling Technology), Mcl-1 (#ab32087, dilution 1/1000, Abcam) and GAPDH (#ab8245, dilution 1/5000; Abcam) were used.

### Luciferase reporter assay

The fragment of wild-type (wt) or mutant (mut) SNHG1 containing the predicted binding site was amplified by PCR, and then cloned into the pmirGLO vector (Promega). After that, miR-101 or control miRNA, together with wt or mut SNHG1, were co-transfected into HEK-293 cells. Finally, luciferase activity was measured using a Dual Luciferase Reporter Assay System (Promega) according to the manufacturer's instructions.

### Animal experiments

Animal experiments were performed as previously described (Sun et al., 2018). Briefly, 8-week-old-male NOD-SCID mice (Beijing Vital River Laboratory Animal Technology Co., Ltd) were randomly divided to three groups. Next, PBS or 5×10^6^ MOLM-13 cells transduced with sh-NC or sh-SNHG1 were transplanted into these mice by tail intravenous injection. At 3 weeks after transplantation, the percentage of GFP+ cells in the BM, spleen and PB of recipient mice were detected by flow cytometry, or *in vivo* Brdu incorporation assay was performed using a APC-BrdU Flow kit (BD Pharmingen, San Diego, CA, USA) according to the manufacturer's instructions. In another experiment, the survival rates of mice were monitored after transplantation. All animal experiments were conducted according to the institutional ethical guidelines for the Care and Use of Laboratory Animals (China Three Gorges University).

### Statistics

All data analysis was performed using SPSS 20.0 and GraphPad Prism 6 software. The differences between groups and among multiple groups were analyzed using the Student's *t*-test and one-way ANOVA, respectively. The correlation between SNHG1 level and clinicopathological characteristics in AML patients was determined using the chi-square test. Kaplan–Meier method and Cox regression models (univariate and multivariate) were conducted to evaluate the effect of SNHG1 expression on overall survival. Spearman’s test was used to analyze the correlation between SNHG1 and miR-101. All experiments were repeated independently at least three times. Data were presented as the mean±s.d. *P*<0.05 was defined as statistically significant.

## Supplementary Material

Supplementary information
