# Supplementary material for: Long non-coding RNA SNHG1 indicates poor prognosis and facilitates disease progression in acute myeloid leukemia
Source: Biol Open. 2019 Oct 15;8(10):bio046417. doi: 10.1242/bio.046417 (PMC6826290; doi:10.1242/bio.046417)
Supplement: Supplementary information [file biolopen-8-046417-s1.pdf]

Table S1. **Clinical characteristics of AML patients using 2016 WHO classification**

Variables	n	SNHG1 expression	
		High (44)	Low (45)
AML with t(8;21)(q22;q22.1)	9	2	7
AML with inv(16)(p13.1q22) or t(16;16)(p13.1;q22)	4	1	3
AML with t(9;11)(p21.3;q23.3)	3	1	2
AML with t(6;9)(p23;q34.1)	4	4	0
AML with inv(3)(q21.3q26.2) or t(3;3)(q21.3;q26.2)	4	1	3
AML with mutated *NPM1*	11	6	5
AML with biallelic mutations of *CEBPA*	12	7	5
AML with myelodysplasia-related changes	14	8	6
AML, NOS			
AML with minimal differentiation	1	1	0
AML without maturation	6	4	2
AML with maturation	13	6	7
Acute myelomonocytic leukemia	5	2	3
Acute monoblastic/monocytic leukemia	2	1	1
Pure erythroid leukemia	1	0	1

NOS, not otherwise specified.

Biology Open • Supplementary information

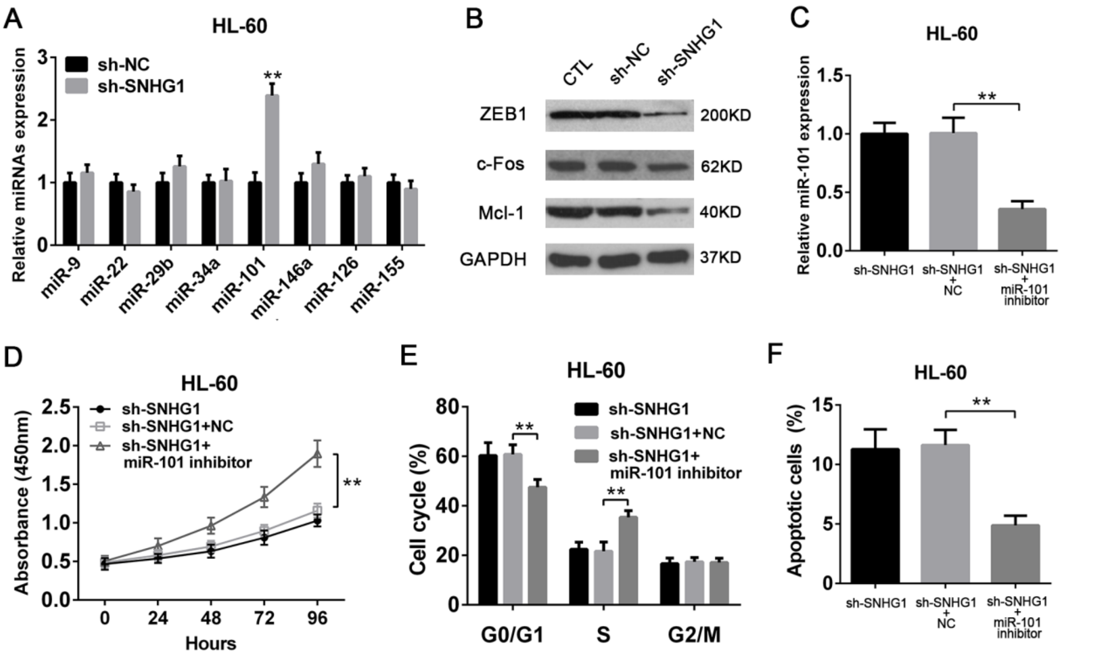

Figure S1. SNHG1 promotes the progression of AML by negatively regulating miR-101. (A) The relative expressions of miR-9, miR-22, miR-29b, miR-34a, miR-101, miR-126, miR-146a and miR-155 in HL-60 cells after knockdown of SNHG1 (n = 6). (B) Western blotting detecting the expressions of c-Fos, ZEB1 and Mcl-1 in HL-60 cells after knockdown of SNHG1. (C-F) HL-60 cells with or without knockdown of SNHG1 were transfected with miR-101 inhibitor or negative control (NC). Then, (C) the expression of miR-101 was analyzed by qPCR. Meanwhile, the (D) proliferation was measured by the CCK-8 assay, and the (E) cell cycle and (F) apoptosis were detected by flow cytometry (n = 6). **p< 0.01.

Biology Open • Supplementary information
